# Recent Advances in Surface Plasmon Resonance Sensors for Sensitive Optical Detection of Pathogens

**DOI:** 10.3390/bios12030180

**Published:** 2022-03-17

**Authors:** Joon-Ha Park, Yeon-Woo Cho, Tae-Hyung Kim

**Affiliations:** School of Integrative Engineering, Chung-Ang University, 84 Heukseuk-ro, Dongjak-gu, Seoul 06974, Korea; joonha95@cau.ac.kr (J.-H.P.); tree7391@cau.ac.kr (Y.-W.C.)

**Keywords:** SPR, optical sensor, graphene oxide, gold, nucleic acid, biosensing techniques

## Abstract

The advancement of science and technology has led to the recent development of highly sensitive pathogen biosensing techniques. The effective treatment of pathogen infections requires sensing technologies to not only be sensitive but also render results in real-time. This review thus summarises the recent advances in optical surface plasmon resonance (SPR) sensor technology, which possesses the aforementioned advantages. Specifically, this technology allows for the detection of specific pathogens by applying nano-sized materials. This review focuses on various nanomaterials that are used to ensure the performance and high selectivity of SPR sensors. This review will undoubtedly accelerate the development of optical biosensing technology, thus allowing for real-time diagnosis and the timely delivery of appropriate treatments as well as preventing the spread of highly contagious pathogens.

## 1. Introduction

Pathogens are defined as foreign antigens that have adverse effects, such as (1) producing toxins, (2) penetrating tissues, (3) colonising tissues, (4) intercepting nutrients, and (5) immunosuppressing the host [1,2,3]. Particularly, contagious pathogens (e.g., those that cause bacterial urinary tract infection, malaria, influenza virus, dengue virus, human immunodeficiency virus (HIV), and severe acute respiratory syndrome coronavirus (SARS-CoV-2) are capable of human-to-human presymptomatic transmission [4,5,6,7,8,9]. Infection pathways with receptor-ligand interactions (RLIs) constitute a representative mechanism through which pathogens bind to their target [10,11,12,13]. Additionally, given that RLIs are biological events that occur in living cells, a high-sensitivity diagnostic sensor is required to detect pathogens [14]. Therefore, biosensing approaches must be constantly optimised to achieve better signal sensitivity.

To increase the sensitivity and selectivity between specific pathogens, recent studies have developed and evaluated novel diagnostic tools that incorporate pathogen binding mechanisms and antigen capture strategies, such as antigen–antibody and aptamer–ligand interactions [15,16]. Current diagnostic procedures are based on several clinicopathological analysis methods, such as gene sequencing, polymerase chain reaction (PCR), fluorescence methods, mass spectrometry, and enzyme-linked immunosorbent assays (ELISA) [17,18,19,20,21,22,23,24]. However, although these bioanalytical methods are highly accurate, each of them has unique limitations. For example, (1) the fluorescence method requires labelling the sample with a fluorescent probe, which adds complexity to the procedure; (2) the analysis of multiple samples using mass spectrometry is highly time-consuming; and (3) most of the aforementioned techniques are generally expensive and require sophisticated equipment and specialised processing, in addition to having a high response time [25,26,27,28,29,30]. To address these limitations, bioanalytical techniques based on optical biosensors have recently garnered increasing attention [31].

Particularly, plasmonic-based biosensors constitute a next-generation technology that facilitates the miniaturisation of biosensors, thereby enhancing detection throughput and reducing operating costs [32]. Pathogen detection can be based on a variety of plasmonic phenomena, including SPR, localized SPR, surface-enhanced Raman scattering, surface-enhanced infrared absorption spectroscopy, and surface-enhanced fluorescence [33]. Among these novel approaches, surface plasmon resonance (SPR)-based sensing techniques are a promising new means to detect pathogens quickly with high sensitivity, and therefore, this method has begun to replace other conventional diagnostic approaches. SPR is an electromagnetic surface wave that propagates parallel to the interface region. On the basis of this principle, changes in a given sample can be detected by monitoring the intensity of the SPR angle (θ) reflected after the plasmon wave of a medium with a low refractive index permeates [34,35]. SPR sensors have some minor disadvantages, such as the possibility of sample SPR peak deflection when a strong electromagnetic field acts on sensitive biological samples [36]. However, there are four main advantages that compensate for these drawbacks: (1) this approach is non-destructive and non-invasive—in other words, it does not cause any damage or physicochemical transformation to the sample; (2) it allows for the sensitive optical quantification of the interaction between molecules in real-time without the need for a separate probe using a radioactive or fluorescent material; (3) after the detection of the target analyte, the surface of the sensor can be reactivated and used repeatedly upon treatment with a regeneration solution; and (4) the lifetimes of nanoparticles can be flexibly controlled and extended through surfactant surface coating or chemical conjugation (e.g., hybrid nanomaterials) [37,38,39,40,41]. Additionally, this approach is rapid, accurate, cost-effective, requires no specialised training, and can be applied to detect a wide variety of targets [42,43,44]. Collectively, these advantages make SPR-based sensing techniques especially well-suited for real-time diagnosis both in clinical laboratories and in the field [45,46,47].

**Figure 1:**
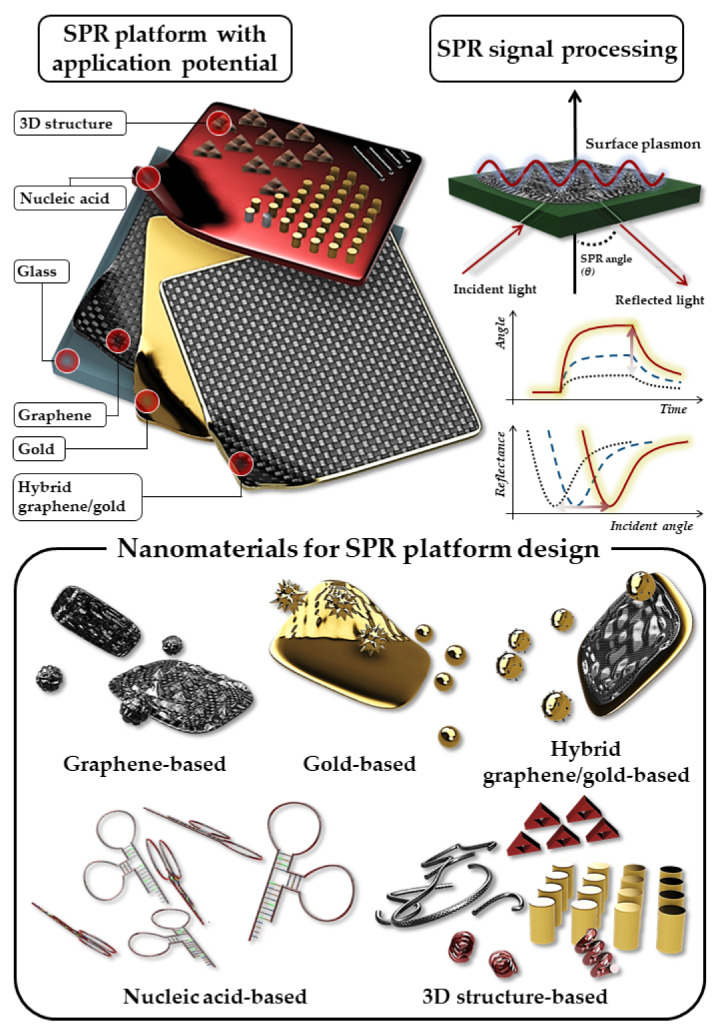
Schematic of recent advances in SPR sensor technology with various nanomaterials and applications for pathogen detection.

SPR sensors based on novel materials are actively being developed. For example, some metals, such as gold film or particle-based SPR platforms, are used to facilitate particle generation and optimise plasmonic performance [48,49,50,51,52,53,54,55]. For instance, graphene-based SPR platforms have excellent analyte adsorption properties, great corrosion resistance, and high thermal resistance [56,57,58,59]. Hybrid graphene/gold-based SPR platforms maximise plasmonic performance via graphene–gold interactions [60,61,62,63,64]. Aptamer-based SPR sensors reportedly exhibit uniquely high analytical specificity [65,66,67,68]. Three-dimensional (3D) structure-based SPR platforms possess a higher surface area than their 2D counterparts, which increases the likelihood of the analyte binding to the platform [69,70,71]. Additionally, these nanomaterials are not only highly biocompatible but also have fewer negative environmental impacts. In fact, some of these materials are actively used for environmental remediation [72,73]. SPR biosensors are therefore often used as a preliminary diagnostic tool, as they enable efficient and high-throughput diagnosis due to their fast detection time and the fact that they do not require labelling [74,75]. In this section, SPR platforms were classified into five categories depending on the materials used for pathogen sensing: (1) graphene oxide-based, (2) gold-based, (3) hybrid graphene/gold-based, (4) nucleic acid-based, and (5) 3D structure-based (Table 1). In this review, different SPR-based sensing techniques that can rapidly detect pathogens, such as viruses and bacteria, are investigated and discussed (Figure 1).

## 2. Graphene Oxide-Based SPR Sensor

The edge of the graphene oxide (GO) structure is rich in oxygen functional groups; it is a single-layer, two-dimensional carbon material [92,93]. GO has outstanding properties that show great potential for biosensing at the molecular level because of its abundant π-conjugation structure along with its large surface-to-volume ratio [94]. Therefore, GO has specific intrinsic features that enable the robust immobilization of biomolecules on the SPR biosensor platform.

**Figure 2:**
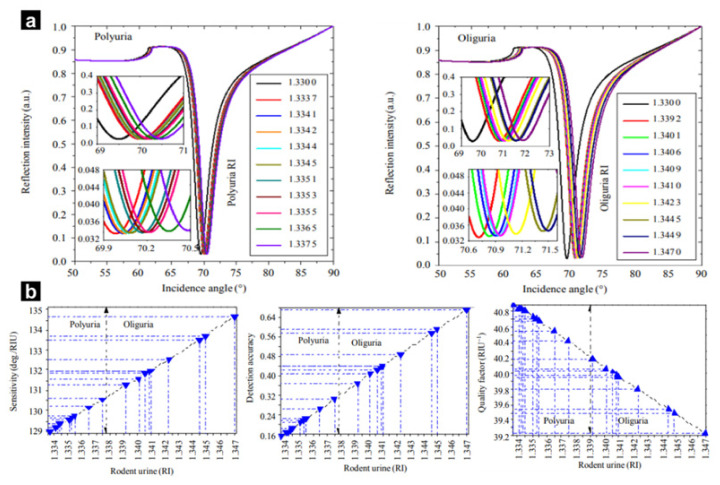
(**a**) SPR signal intensity vs. incident angle plot with polyuria (left) and oliguria (right). (**b**) Variation in parameters for different sensing medium refractive indices in pure water, polyuria, and oliguria. With permission from [76], Copyright 2020, Springer.

A recent study reported the detection of Leptospirosis bacteria using a graphene-based SPR sensor [76]. The researchers coated a glass platform with graphene to effectively adsorb the bacteria. Rodent urea was then used as the main sample, and the analytical outcomes were classified as polyuria (high urine production) and oliguria (low urine production). Upon measuring the urea with the SPR platform, polyuria was defined between 128.8378 (deg/RIU) and 130.40 (deg/RIU), whereas oliguria was defined between 134.5118 (deg/RIU) and 131.0978 (deg/RIU) with different angles of reflection (Figure 2a). The proposed graphene-based SPR sensor has been theoretically and numerically analysed to detect Leptospirosis-causing bacteria in rodent urine, suggesting that it can be utilised for the early detection of this disease with high sensitivity and accuracy (Figure 2b). Although graphene can be used to increase SPR signals, additional efforts have been made to improve sensitivity and detection accuracy. To achieve this, researchers have begun to study barium titanate nanocomposites. Through the adsorption of barium titanate onto graphene, the proposed sensor achieved a higher detection sensitivity compared to a single graphene-based SPR biosensor. Furthermore, Z. Xia et al. proposed an SPR sensor based on barium titanate and also achieved high analyte sensitivity [95]. Another study detected *Pseudomonas* bacteria by mixing graphene and barium titanate [77]. The platform was fabricated by deposition of silver and graphene on a BK7 prism glass plate that refracts incident light, and the sensors were constructed in the presence or absence of barium titanate (BaTiO_3_) nanocomposites. The authors reported that the maximum sensitivity, detection accuracy, and quality parameters of the sensor were 220 deg/RIU, 7.09, and 101.38 RIU^−1^, respectively. Therefore, this SPR platform can sensitively detect *Pseudomonas* bacteria. Additionally, the authors demonstrated that the graphene thickness had a critical effect on the results even when the affinity of the layers changed, and the monolayer exhibited the best reflectivity curve.

Quantum dots are a nanomaterial with a high surface area and signal-to-noise ratio, and significantly improved data accuracy [96]. Additionally, combining graphene oxide with carboxyl functional groups and quantum dots greatly increases mechanical stability as well as the potential for SPR signal amplification [97]. A study from 2019 reported the detection of dengue virus E-protein by adsorbing cadmium sulphide quantum dots on an optical SPR platform based on graphene oxide [78]. The SPR platform with cadmium sulphide quantum dots was fabricated through the stirring method, and the matrix of the sensor was based on graphene oxide. The fabricated SPR platform achieved a detection limit of 0.001 nM for the dengue virus E-protein, which demonstrated its high sensitivity, and the estimable linear range of the sensor was 0.0001 to 0.01 nM. These sensing results suggest that graphene oxide can improve the performance of SPR biosensors and is thus a promising candidate in the field of biosensing.

The above-described observations demonstrate that graphene oxide-based materials have various advantages for pathogen detection. It has been proven that GO does not damage biological samples, nor does it interfere with the SPR effect. Moreover, because of the characteristics of GO, such as its easy handling and excellent analyte adsorption properties, highly sensitive GO-based SPR sensors are expected to be developed in the future.

## 3. Gold-Based SPR Sensor

Gold-based materials can be easily synthesised and are highly biocompatible. Additionally, these materials are structurally strong, and their optical properties can be fine-tuned depending on the conditions of the platform. Therefore, gold is widely used in biosensing chips [98,99]. Over the past decade, gold has been applied in various forms, and previous studies have demonstrated that this material exhibits strong red-shifting properties [100]. Furthermore, the strong radioactive properties of gold particles, such as absorption, scattering, and plasmonic field generation, can be widely applied to SPR biochips.

Bong et al. developed a gold-based SPR sensor for SARS-CoV-2 [79]. The sensor was fabricated by binding antibodies through chemical conjugation on the gold chip sensor, which was the base of this SPR platform. The limit of detection for an analyte isolated from serum was 1.02 pM (i.e., the sensor was highly sensitive). Additionally, the sensor only detected coronavirus and no other influenza viruses, thus demonstrating its high selectivity for the analyte due to the characteristics of the substrate. Moreover, the gold-based SPR sensor rendered results very quickly, making this approach uniquely well-suited for the accurate detection of diseases at an early stage.

Spherical gold nanoparticles have various advantages compared to gold films or random gold particles, such as their narrow size distribution, consistent shape and size, and easy handling. Particularly, the size specificity of gold nanoparticles results in a narrow standard deviation from the analyte signal during SPR measurement. Furthermore, antibodies that specifically recognise various chemical conjugates or analytes can be attached to the spherical particles, thereby taking advantage of the large surface area of the nanoparticles and enhancing the SPR signal. A study from 2017 reported the development of a surface plasmon sensor using an antibody on a gold-based platform to sense pathogenic bacteria [80]. The fabricated biosensor was based on the principle of strengthening the surface plasmon effect by adding gold nanoparticles (AuNPs) after binding the analyte on the gold sensor chip. The limit of detection for the analytes of this fabricated SPR platform was 8 × 10^6^ CFU/mL, and the sensor was specifically sensitive to the pathogenic bacterium *Campylobacter jejuni*. Furthermore, the AuNPs in the sensor increased the efficiency of analyte analysis, whereas the gold sensor chip enhanced SPR sensitivity. Interestingly, although the use of AuNPs enhanced SPR performance, the SPR effect was maximised when using other types of gold nanostructures, such as nano-urchins or nanospikes.

**Figure 3:**
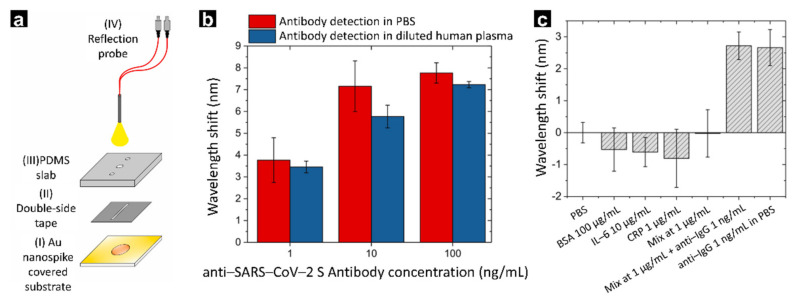
(**a**) Schematic illustration of the fabricated gold nanospike-based SPR sensor. (**b**) Plot representing the detection limit for the analyte. (**c**) Selective affinity test against other interference substances. With permission from [81], Copyright 2020, ELSEVIER.

Similar to sea urchins, gold nanospikes are not perfectly circular but have a sharp and uneven surface. This unique structure could enhance the red-shifting of the surface plasmon resonance absorption peak, in addition to increasing the electromagnetic field at the tip of the spike structure compared to that of a spherical particle. A recent study developed a gold spike nanoparticle-based SPR sensor that detects SARS-CoV-2 in human plasma [81]. The researchers deposited the gold spike nanoparticles through electrodeposition onto a glass slide rather than gold in its bulk state (Figure 3a). The fabricated SPR sensor exhibited a low limit of detection of 0.08 ng/mL (~0.5 pM) for SARS-CoV-2 in addition to being highly specific, as it did not react to three competing substances and BSA protein (Figure 3b,c). This shows that the fabricated SPR sensor could be used for the early diagnosis of COVID-19 and can accurately recognise virus analytes without detecting other substances remaining in the human plasma.

Studies have demonstrated that bimetallic combinations can provide greater sensitivity, signal-to-noise ratio (SNR), and increased operating range values, all of which cannot be achieved with a single metal layer. A recent study sought to offset the shortcomings of single metal layer-based sensors and improve the performance of the SPR sensor by using a novel bimetal layer structure of Au and aluminium (Al) [82]. The researchers detected the DENV2 NS1 protein with the developed SPR sensor by laying a gold and aluminium bilayer on the SU-8 spacer. The fabricated SPR sensor recorded a limit of detection of 0.1 μg/mL for DENV2-NS1, and the linear range of the sensor was between 0.1 and 10 μg/mL. Interestingly, the limits of detection in both PBS and blood plasma were also within a similar range (i.e., as low as 0.1 µg/mL), suggesting that this approach can detect viruses directly in blood samples or liquid biopsies. This study demonstrated that a specific virus can be detected with high sensitivity through immediate screening in clinical samples.

According to a recent study, on-chip integration of electronics and plasmonics would allow for samples to be guided to relevant locations and sensed thereafter [101,102,103,104]. Particularly, gold-based plasmonic trapping technology could substantially improve the efficiency of broadband photodetectors and SPR sensors [105,106]. Gold nanostructures have a virtually infinite potential depending on how they are applied. These structures can be constructed using various methods, such as growing gold on a substrate, attaching it by chemical conjugation, or depositing it. Similarly, the ability of the sensor to detect the analyte can be fine-tuned. The implementation of gold in the SPR sensor enhances its surface plasmon effect, the most basic characteristic of metal, and causes no damage to the platform body, both of which are considered crucial advantages of this material.

## 4. Hybrid Graphene/Gold-Based SPR Sensor

Gold-based biosensors are currently preferred over other sensors based on other metals. However, according to a recent study, there is a risk of peeling if the gold layer coating on the platform surface becomes too thin [107]. Furthermore, another study reported that gold itself has little influence on the adsorption of biomolecules, which can lower the detection limit [108]. Additionally, sensors based only on graphene exhibit an excellent adsorption force for the detection of analytes, but their surface plasmonic effect is significantly lower than that of metal-based sensors [109]. To compensate for this limitation, several recent studies have developed biosensors based on a hybrid graphene/gold structure.

**Figure 4:**
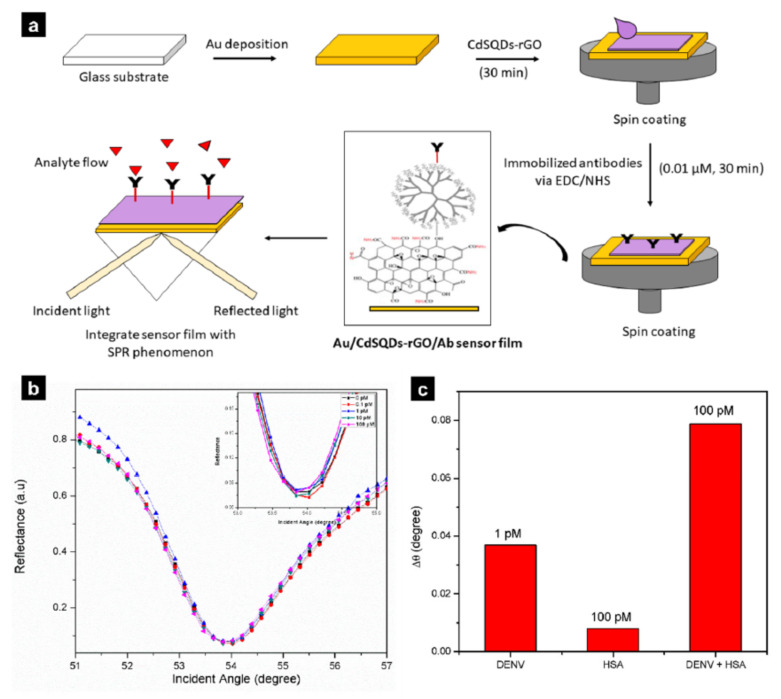
(**a**) Schematic illustration of the biosensor fabrication process. (**b**) Plot indicating the limit of detection and linear range. (**c**) Selectivity of the fabricated platform. With permission from [83], Copyright 2019, MDPI.

Omar, N.A.S. et al. developed a hybrid graphene/gold sensor to detect the dengue virus by thinly coating reduced graphene on a gold film [83]. The generated SPR platform was coated with gold in the form of a film through deposition on a glass substrate, and graphene was thinly coated through spin-coating (Figure 4a). This hybrid sensor achieved a detection limit of 28 fM and a linear range of 0 pM to 10 pM, as determined with a dengue virus solution (Figure 4b). A sample of 100 pM human serum albumin (HSA) was used for the selectivity test, and the fabricated platform reacted only with the dengue virus and showed low reactivity to HSA (Figure 4c). These results demonstrated that the hybrid graphene/gold SPR sensor could potentially be used in health and environmental monitoring. In addition to gold films, gold nanoparticles, an important type of plasmon nanoparticles, possess excellent plasmonic properties and have thus begun to be implemented in a variety of applications. The use of particles maximises the surface area of the gold film and graphene oxide complex. At the same time, the spherical gold nanoparticles adsorb to the graphene to enhance adsorption and plasmonic performance [84]. An SPR sensor was developed by combining graphene oxide and spherical gold nanoparticles through sonication and stirring. The researchers detected A-type and O-type foot-and-mouth disease viral particles through the resulting substrate and achieved limits of detection of 100 fg/mL and 100 pg/mL, respectively. Because of the characteristics of the GO on the substrate, single-stranded DNA (ssDNA) can readily bind to the sensor through electrostatic interactions, and thus the limit of detection is 1000 times lower than that of conventional PCR. Therefore, combining pristine gold nanoparticles and GO can greatly improve the limit of detection of biosensors.

Many SPR sensors have been developed to detect analytes by adsorbing gold nanoparticles on graphene; however, this method is notorious for its limitations. Specifically, a recent study reported that the performance of the sensor was low because the adsorbed gold nanoparticles tend to agglomerate on the platform surface, and the absorbance curve is widened, thereby decreasing sensor stability [110]. To solve this problem, recent studies have explored the encapsulation of gold nanoparticles using graphene. For example, a related study used GO to encapsulate gold nanorods, thereby overcoming the aforementioned limitations [85]. Concretely, the researchers developed a GO-encapsulated gold nanorod-based SPR sensor to detect hepatitis B, and this platform was fabricated through sulphonylation. To increase the specificity of the sensor towards hepatitis B, HBsAg antibodies were decorated on the GO surface. The limit of detection for hepatitis B was 0.05 pg/mL, and the sensor had a wide linear range of 1–1000 pg/mL. The researchers then diagnosed HBV-infected patients with the fabricated platform and achieved a relative standard deviation (RSD) of less than 5%, thus demonstrating the potential applicability of the sensor in clinical diagnosis.

A hybrid platform decorated with a gold film or nanoparticles on graphene oxide showed the potential to enhance the surface plasmon resonance performance of the sensor. Additionally, by taking advantage of the properties of GO, an antibody that can specifically recognise an analyte can be adsorbed on the surface, thus allowing for the biosensor to be used alongside immunoassays. This type of sensor is much more versatile because it combines the advantages of GO and gold. Additionally, sensing performance is substantially higher than that of sensors based on GO or gold alone. Nevertheless, additional studies are required to better understand this hybrid technique and which factors can be modified to optimise its performance.

## 5. Nucleic Acid-Based SPR Sensor

Aptamer-based biosensors have recently garnered increasing attention because they integrate the advantages of antibody-based sensors while also exhibiting excellent thermal stability, low cost, and an extremely wide variety of applications [111,112,113]. Furthermore, unlike antibodies, nucleic acid ligand aptamers can be chemically synthesised and easily modified using functional groups, in addition to being highly stable [114]. When this biomaterial binds to an analyte of interest, it has a similar or stronger binding force than that of antibodies, thus enabling stable sensitivity measurements [115]. Because of these characteristics, aptamers can be widely applied in the fields of biosensors, diagnostics, and research agents.

A study conducted in 2018 reported the detection of virulence factors using an aptamer-based SPR sensor [86]. The SPR sensor was fabricated by binding the target protein on a gold film through various chemical conjugation steps. The aptamer was selected to be specific to the analyte through the systematic evolution of ligands via the exponential enrichment (SELEX) technique. The sensor exhibited a low limit of detection for *Shigella sonnei*, and the measurable linear range was 0–100 nm/mL. Except for *S. sonnei*, all other *Shigella* and non-*Shigella* species exhibited a low recognition rate, thus demonstrating the high specificity of the sensor. This is an example of aptamer selection through SELEX, which substantially enhanced the analyte selectivity for a specific pathogen species. Specifically, the incorporation of aptamers dramatically increased the selectivity to the analyte, but the approach was not sufficiently sensitive. To solve this problem, the authors proposed a method to enhance sensitivity by attaching a pair of aptamers to a specific analyte.

A sensor with a pair of aptamers in a sandwich configuration was reported to improve the sensitivity and specificity on various platforms. Therefore, this approach has recently been implemented to optimise SPR biosensors. The use of a secondary aptamer amplifies the signal, and these types of biosensors are suitable for fast diagnosis because they allow for the real-time monitoring of any signal changes. A recent study employed this sandwich aptamer technique to construct an SPR sensor to detect pathogens [87]. The fabricated SPR platform consisted of an aptamer attached to a paper strip sensor. The sensor became red upon contact with the analyte, and the colour shift could be quantified using the SPR principle. The limit of detection for the analyte was 10^3^–10^4^ CFU/mL, and the sensor had a useful analytical range of 4 × 10^1^–4 × 10^5^ CFU/mL. Moreover, because of its aptamer properties, the sensor exhibited low specificity for other analytes, except for *Vibrio fischeri*. The authors thus concluded that this paper strip-based sensor could be used for the real-time detection of analytes in the field.

**Figure 5:**
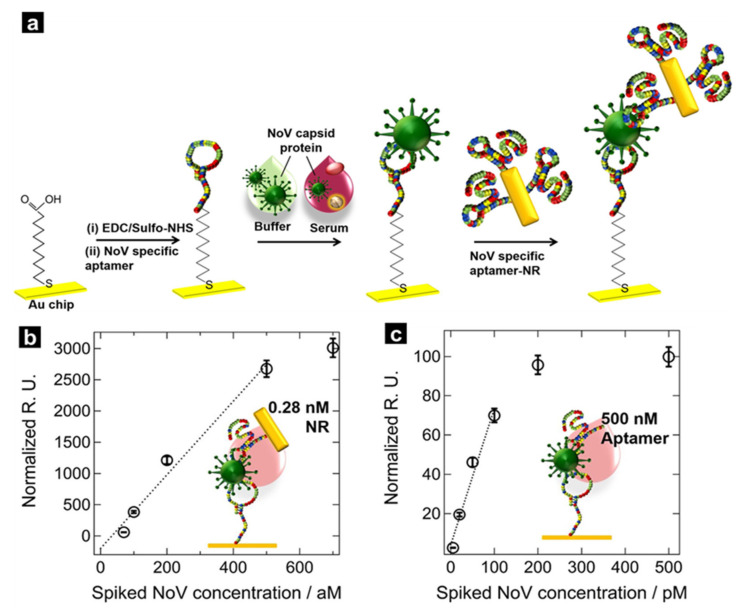
(**a**) Schematic illustration of SPR sensor fabrication via the sandwich aptamer technique. (**b**) Linear plot of norovirus spike protein-sensing performance. (**c**) Linear plot of norovirus spike protein-sensing performance without gold nanorods. With permission from [88], Copyright 2018, American Chemical Society.

Although the sandwich aptamer technique can achieve high levels of sensitivity, its plasmonic effect could be further improved. Therefore, studies on the binding of metals to the secondary aptamer have been actively conducted, and a recent study demonstrated that the SPR effect could be improved by binding gold nanoparticles [88]. The researchers connected the primary aptamer to a gold chip through chemical conjugation, after which the norovirus capsid protein was bound (Figure 5a). The secondary aptamer was bound to the analyte while attached to gold nanorods, which were fabricated through the seed-growth method. The limit of detection of the generated SPR sensor was approximately 70 aM, thus demonstrating that the sensor was very sensitive to the analyte (Figure 5b). Notably, the signal was 10^5^ times stronger than when gold nanorods were not used (Figure 5c). Additionally, by using two aptamers as capturing reagents for the norovirus capsid protein, non-specific binding events of other substances could be minimised. The aptamer–aptamer surface sandwich SPR platform with nanorod amplification is a promising means for the direct analysis of difficult-to-detect virus samples, and a wider variety of analyte-specific aptamers is expected to be developed in the future.

Prior to the application of aptamers to the SPR sensor, this technology developed rather slowly. However, aptamer-based sensors have been stably developed over the past decade to this day. Aptamers are commonly selected by SELEX, but this technique is known for its limitations in targeting pathogens. This is because the highly variable and complex structure of the pathogen can affect the performance of the aptamer. Therefore, a simpler and more efficient SELEX method must be developed to facilitate the synthesis of universal aptamers for various pathogens. Aptamer–analyte capturing methods have already been developed, and the limitations of existing aptamers can almost be fully overcome by binding metals to the secondary aptamer, among other strategies. Nevertheless, additional efforts are needed to broaden the applicability of these promising sensing technologies while also solving their limitations.

## 6. 3D Structure-BASED SPR Sensor

Simple two-dimensional structures greatly limit the application of the plasmonic effect for pathogen sensing. Additionally, the attachment of chemical conjugates, aptamers, and antibodies significantly affects the surface area of the sensor, which has been a major obstacle for platform development [116]. In response to these limitations, 3D structures began to be implemented in biosensors, particularly to increase surface area. Moreover, the three-dimensional nature of these structures enhances the electromagnetic field at the interface between the structural gaps, which improves pathogen detection.

A recent study successfully detected the Epstein-Barr virus (EBV) using this 3D structure approach [89]. The SPR 3D structure sensor developed by the researchers was made through nanoimprinting lithography, and a nanowire-type platform was constructed via gold deposition. The limit of detection for the analyte was 4.1 × 10^−5^ RIU, and the sensor exhibited high specificity. The developed 500 nm periodic nanowire 3D structure SPR sensor could successfully detect DNA and genes without labelling. Furthermore, the detection system developed in this study was not only cost-effective but also had excellent measurement and production repeatability.

To further maximise the efficiency of the electromagnetic field between the 3D-shaped structures, researchers have developed a platform in which certain shapes are tightly packed. This approach not only increases the surface area further but also enhances the SPR signal efficiency for pathogen detection. For example, a recent study reported the detection of rotavirus via the application of an octupolar nanopattern to a platform [90]. The researchers formed gold nano prisms on the sensor with an electron-beam lithography (EBL) system, which was based on octupolar geometry. The limit of detection of the fabricated platform was 126 ± 3 PFU/mL, which was lower than the plaque-forming unit (1 × 10^3^ PFU/mL) of rotavirus in water, thus demonstrating a sensitive detection potential. To measure the selectivity of the generated platform, the researchers measured bovine herpesvirus (BHV1) and equine viral arteritis (EVA), neither of which elicited a response from the sensor. In this way, the disadvantages of the existing method could be overcome, and current findings suggest that efficient pathogen detection can be cost-effectively achieved using portable point-of-care approaches.

**Figure 6:**
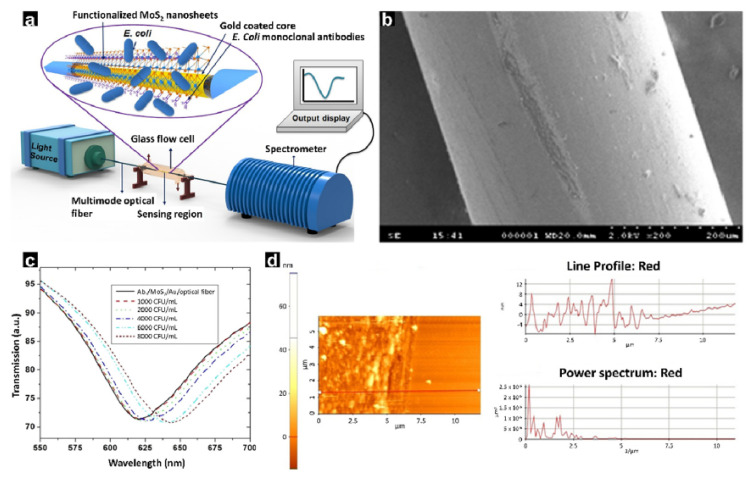
(**a**) Schematic illustration of an experimental setup to test sensors. (**b**) Field emission scanning electron microscope (FESEM) image of the fabricated optic fibre surface. (**c**) Transmission spectra plot indicating the limit of detection. (**d**) Atomic force microscope image analysis of the fabricated optic fibre surface. With permission from [91], Copyright 2018, Elsevier.

In recent years, many 3D optical fibre immunosensors based on surface modification using nanomaterials have been reported to improve biocompatibility [117,118,119]. Additionally, SPR sensors combining 2D material nanosheets and 3D fibres have been recently developed. Particularly, the properties of 2D MoS_2_ nanosheets have attracted considerable interest from the scientific community because of their broad applicability in the field of biosensing [120]. This combination has significant advantages: (1) high electron mobility, (2) low toxicity, (3) high surface area, and (4) thermal stability. A recent study reported a 3D fibre optic SPR sensor for quantitative analysis of *E. coli* (Figure 6a) [91]. An optical fibre SPR sensor was developed through ultrasonication and etching, after which MoS_2_ was coated on the cylindrical fibre surface (Figure 6b). The limit of detection of the fabricated SPR platform was 94 CFU/mL (Figure 6c), and the sensor exhibited excellent selectivity for *E. coli* because cross-reaction with other materials was prevented by the MoS_2_ coated on the surface (Figure 6d). This 3D optic fibre SPR sensor exhibited significantly higher sensitivity and in vivo functionalization compared to other similar sensors, in addition to rendering rapid results. However, the instability of the antibody increased after a certain amount of time, and defects in the surface or edge of the MoS_2_ nanosheets caused sulphur vacancies. Therefore, more studies are needed to improve the stability and robustness of the sensor.

Interestingly, the reported 3D structure-based SPR sensor exhibited a high surface area, excellent electromagnetic field efficacy, and SPR signal enhancement ability due to its material and structural properties. Particularly, the sensor was able to detect pathogens in real-time in the field with a lower limit of detection compared to conventional 2D materials alone. It has been proven that the combination of 3D structures and 2D materials not only enhances in vivo functionalization but can also maximise SPR-sensing performance. By taking advantage of fact that the results differ depending on how the structures and materials are utilised, 3D-based sensors can be excellent candidates for the effective detection of a wide variety of targets.

## 7. Conclusions

This review summarised recent research on optical SPR platforms for pathogen detection via the application of different materials. All the different nanomaterials reported above have been proven to exhibit excellent efficacy for pathogen detection. In the case of aptamer-based SPR sensors, rather than simply performing analysis with one aptamer, the sandwich aptamer technique was implemented, and gold nanorods were combined with a secondary aptamer to improve the detection limit of difficult-to-analyse pathogens. Platforms with a 3D nanostructure increased the effectiveness of the electromagnetic field through the arrangement of uniform structures at a high density. The 3D nanostructure led to a strong enhancement of the SPR signal, and these features allow for the rapid detection of pathogens in the field. Therefore, each nanomaterial has its own unique optical properties and various reaction patterns to analytes, and it is expected that the sensing potential of SPR sensors can be maximised by offsetting their disadvantages through the fusion of these materials or the synthesis of novel alternatives.

Increasing numbers of human diseases are reported each year. Therefore, researchers need to develop a sensor that can easily detect different kinds of pathogens. In many situations, the analytes of interest are present in low concentrations in complex media. Therefore, additional efforts are required to improve the materials and technology on which biosensors rely to improve their sensitivity, accuracy, and specificity. Furthermore, the sensing performance of platforms with complex structures is often superior, but this commonly reduces stability. Therefore, in addition to enhancing detection potential, efforts must be made to improve or at least preserve the stability, robustness, and storability of biosensors, as well as to avoid any potential problems when they are used in the field.

## Figures and Tables

**Table 1:** Materials used for the construction of SPR sensors for the optical detection of pathogens.

Materials	Pathogen/Disease	LOD	Ref.
Graphene oxide film	Leptospirosis	-	[76]
BaTiO_3_-adsorbed graphene oxide	*Pseudomonas*	220 deg/RIU	[77]
Cadmium sulphide quantum dot-adsorbed graphene oxide	Dengue virus E-protein	0.001 nM	[78]
Gold film	SARS-CoV-2	1.02 pM	[79]
Antibody-conjugated gold nanoparticles	*Campylobacter jejuni*	8 × 10^6^ CFU/mL	[80]
Gold nanospikes	SARS-CoV-2	0.5 pM	[81]
Gold–aluminium bilayer	DENV2-NS1	0.1 μg/mL	[82]
Hybrid graphene/gold film	Dengue virus	28 fM	[83]
Spherical gold nanoparticles on graphene oxide film	A and O-type foot-and-mouth disease virus	A-types: 100 fg/mL O-types: 100 pg/mL	[84]
Graphene-encapsulated gold nanoparticles	Hepatitis B	0.05 pg/mL	[85]
Aptamer on gold film	*Shigella sonnei*	-	[86]
Aptamer–aptamer sandwich formation	*Vibrio fischeri*	10^3^–10^4^ CFU/mL	[87]
Sandwich formation with gold nanorods	Norovirus capsid protein	70 aM	[88]
Gold nanowire-type	Epstein-Barr virus	4.1 × 10^−5^ RIU	[89]
Gold nanoprisms	Rotavirus	126 ± 3 PFU/mL	[90]
MoS_2_-coated gold optical fibre	*Escherichia coli*	94 CFU/mL	[91]

## Data Availability

Not applicable.
